# Familial Hypertrophic Cardiomyopathy: Late Potentials and Other Prognostic Markers

**DOI:** 10.7759/cureus.6530

**Published:** 2020-01-01

**Authors:** Ândrea Chaves-Markman, Manuel Markman, Marcelo Antônio O Santos-Veloso, Lucas S Bezerra, Dário C Sobral Filho, Brivaldo Markman Filho

**Affiliations:** 1 Cardiology, Rarus - A Rare Disease Service, Recife, BES; 2 Echocardiography, Agamenon Magalhães Hospital, Recife, BRA; 3 Internal Medicine, Hospital dos Servidores do Estado, Recife, BRA; 4 Miscellaneous, Maurício de Nassau University, Recife, BRA; 5 Cardiology, Faculty of Medical Sciences, University of Pernambuco, Recife, BRA; 6 Cardiology, Federal University of Pernambuco, Recife, BRA

**Keywords:** familial hypertrophic cardiomyopathy, sudden death, cardiogenic syncope, late potentials, prognostic marker

## Abstract

Familial hypertrophic cardiomyopathy is an autosomal dominant genetic disease considered the most common cause of sudden cardiac death in individuals under 35 years old, especially the athletes. This study aimed to investigate the association between the presence of late potentials and a family history of sudden death, syncope, and complex ventricular arrhythmias on patients with hypertrophic cardiomyopathy. A case series study was carried out from March 2001 to December 2002, including 22 patients with hypertrophic cardiomyopathy according to transthoracic echocardiogram criteria. Patients on a cardiac pacemaker, right bundle branch block, cardiac transplant, and under no possibilities to realize the exams were excluded. The results showed that asymmetric septal hypertrophy was the most common type (73%), 63% had a positive familial history of hypertrophic cardiomyopathy, 55% sudden cardiac death, and 23% syncope. Also, complex ventricular arrhythmias were detected in 14% and late potentials in 23% of patients. According to this study, the presence of late potentials was not associated with familial sudden death, syncope, and complex ventricular arrhythmias.

## Introduction

Hypertrophic cardiomyopathy (HCM) is an uncommon cardiac condition that has a 1:500 population prevalence and is the most common cause of sudden death in the young, especially in the athletes [1-2]. The clinical presentation is usually asymptomatic, but symptoms such as dyspnea, syncope, arrhythmias, and sudden cardiac death can be found [1].

HCM presents an important familial characteristic. Even considering that ethnic and racial differences can affect the familial history and genetic profile of the affected subjects, around 1,500 mutations in at least 20 genes encoding the myofilaments of the sarcomere or Z-disc have been studied and associated to this condition [3-6].

The histopathological features include myocytes disarray with disorganized sarcomeric alignment, abnormalities in the coronary arteriolar walls, diastolic dysfunction, elongation, or malformation of mitral valve leaflets and presence of myocardial fibrosis [5,7-9]. HCM is also related to other medical conditions such as Friedreich’s ataxia, Noonan’s syndrome, and some metabolic disorders, for example, amyloidosis, Pompe, and Fabry’s disease [7].

The primary method to evaluate and diagnose HCM is echocardiography, which provides the characterization of diastolic and systolic function, intraventricular obstruction, and valvular abnormalities [8]. HCM diagnosis is made considering the presence of unexplained left ventricular wall thickness ≥ 15mm in one or more myocardial segments or equivalent to body surface mass in children, and ≥13 mm in patients with a positive family history of HCM [6-8].

As a complementary exam, the cardiac magnetic resonance (CMR) provides a better analysis of the morphology, function, and tissue characterization, being mainly used to evaluate the presence of fibrosis as well as morphological abnormalities on the mitral valve (MV) apparatus, especially elongation of the MV leaflets [6,9]. The presence of necrosis and fibrosis seems to reflect on elevated levels of cardiac troponin T and markers of myocardial inflammation, and the existence of an apical aneurysm points to a poor prognosis, increasing the risk for heart failure and sudden arrhythmic death [6-7,10].

## Materials and methods

A retrospective case series was performed from March 2001 to December 2002 at the Federal University of Pernambuco Clinics Hospital (HC/UFPE). The diagnosis was based on the identification of hypertrophic ventricle (wall thickness ≥ 15 mm in adults and the equivalent for the body surface mass in children) in the absence of another cardiac or systemic disease capable of explaining the cardiac hypertrophy [7-8].

The inclusion criteria were HCM diagnosis, according to a two-dimensional transthoracic echocardiogram. Those subjects with a cardiac pacemaker, right bundle branch block, and cardiac transplant were excluded. Initially, 32 patients were selected following the two-dimensional transthoracic echocardiogram criteria for hypertrophic cardiomyopathy (HCM).

Data were collected from medical records, including a family history of HCM and sudden death, history of syncope, 24-h Holter, and high-resolution electrocardiography results. From the initial sample, 10 patients were excluded: three presented right bundle branch block; two had cardiac pacemaker implant; two did not follow-up the consults; one was submitted to cardiac transplant; one did not collect the necessary exams due to technical issues. A total of 22 patients were included in the final analysis.

Definition of sudden death

Sudden death was defined as witnessed acute collapse, which has taken place within one hour from the onset of symptoms and signs of disease in previously healthy subjects or unexpected non-witnessed death during sleep.

Dynamic electrocardiography (24-h Holter)

A continuum digital recorder (Cardio Light®, Cardio Sistemas Commercial Industrial, São Paulo, Brazil) was used to record and analyze the Holter tests in 24 h. The electrodes were positioned in the electrocardiographic derivations CM1, CM5, and modified D2. The results were analyzed in the DMI Burdick analyzer® (Mortara Instruments, Wisconsin, USA) software by an independent observer searching for complex cardiac arrhythmias.

High-resolution electrocardiography

A multivariate digital cardiograph (Multicardiógrafo Digital Cardio Flash®, Cardio Sistemas Comercial Industrial, São Paulo, Brazil) was used to record the high-resolution electrocardiogram for continuous 16 minutes. The electrodes were positioned in the orthogonal bipolar derivations X, Y, and Z. A 40-Hz filter was applied. The results were analyzed in the DMI Burdick analyzer® (Mortara Instruments, Wisconsin, USA). A positive result was defined as the presence of at least two of the following parameters: a) filtered QRS durations > 144ms; b) QRS terminal voltage <20 µV in the last 40 ms; and c) late potential total duration > 38 ms. In complete left branch bundle blockage, the reference values adopted for the QRS terminal voltage and the late potential duration were 17 µV and 55 ms, respectively.

Statistical analyses

Data were analyzed descriptively using the Statistical Package for the Social Sciences version 20.0 (IBM Company, Armonk, NY, USA). Continuous variables were presented with measures of central tendency and dispersion (mean and standard deviation), and categorical variables were described as absolute (n) and relative (%) frequencies. Fisher's test was used to identify any correlations between late potential, family sudden death, history syncope, and complex ventricular arrhythmias as the distributions did not meet the criteria for the *X*² test. A significance level of 0.05 was adopted to reject the null hypothesis.

Ethical standards

The study was approved by the Institutional Ethics Committee and conformed to the Resolution 466/12 of the Brazilian National Health Council, which deals with the guidelines and standards for research involving humans. The investigation was also conducted in accordance with the Declaration of Helsinki. Informed consent was not necessary as the data were collected from medical records, and there was no direct contact between researchers and subjects.

## Results

In this study, we analyzed 22 patients with HMC. Their ages ranged from 7 to 54 years (mean: 25.6 ± 14.7 years), and 55% (n = 12) were women. Clinical characteristics of the sample, including symptoms and imaging exam results, are available in Table 1.

**Table 1:** Sample characteristics A: age; S: sex; M: male; F: female; DS: dyspnea; PP: precordial pain; P: palpitation; Scp: syncope; FH: familial history; SD: sudden death; Med: medications; AM: amiodaron; BB: beta-blocker; BBC: calcium channel blocker; PE: physical exam; ECG: electrocardiography; LVO: left ventricular overload; LAO: left atrial overload; BO: biventricular overload; LBB: left bundle branch block; BAO: biatrial overload; AVB1st degree: first-degree atrioventricular block; VA: complex ventricular arrhythmia; LP: late potentials; ASH: asymmetric septal hypertrophy; RV: right ventricle; CH: concentric hypertrophy; MVH: mid-ventricular hypertrophy

Pt	A	S	DS	PP	P	Scp	FH	FH/SD	Med	PE	ECG	VA	LP	Hypertrophy	Obstruction
1	53	M	-	+	+	-	-	-	AM	Normal	LVO	-	-	ASH	-
2	39	F	+	+	-	+	+	+	-	Normal	LAO.LVO	-	-	ASH	-
3	15	F	+	+	-	-	+	+	-	Normal	LAO.BO	-	-	ASH	-
4	17	F	+	-	-	-	+	+	-	Normal	LAO.LVO	-	+	ASH	-
5	12	F	+	-	-	-	-	-	-	Normal	LVO	-	-	ASH	-
6	45	F	+	-	+	+	+	+	-	Abnormal	LBB	+	+	ASH	-
7	53	M	-	-	-	-	-	-	Others	Normal	LVO	-	-	ASH	-
8	22	M	-	-	-	-	+	-	-	Normal	BAO.BO	+	-	ASH+RV	-
9	32	F	-	+	-	-	+	+	-	Abnormal	BAO. BO	-	+	ASH	-
10	15	F	+	-	-	+	+	-	-	Abnormal	LVO	-	+	ASH	-
11	23	M	-	+	-	-	-	-	BB	Abnormal	LVO	-	-	ASH	-
12	29	M	+	-	-	-	+	+	BBC	Normal	LBB.LVO	-	-	ASH	-
13	13	M	-	+	-	-	-	-	-	Normal	LVO	-	-	ASH	-
14	12	F	-	+	-	-	-	-	BBC	Abnormal	LVO	-	-	CH	-
15	28	M	-	+	-	-	-	-	BB	Normal	LVO	-	-	CH	-
16	51	F	+	-	-	-	+	+	BB	Abnormal	LVO	-	-	ASH	+
17	27	F		+	+	-	+	+	BB	Normal	LVO	-	-	ASH	-
18	38	M	+	+	+	+	+	+	BB	Normal	LVO	+	-	ASH	-
19	54	M	-	-	-	-	-	-	BB	Normal	LBB	-	+	MVH	-
20	21	F	+	+	-	+	+	+	BBC. AM	Abnormal	LAO. BO AVB1st degree	-	-	MVH	+
21	7	F	-	-	-	-	+	+	BBC	Abnormal	LAO.LVO.	-	-	MVH	+
22	11	M	+	-	-	-	+	+	BBC	Abnormal	LVO.LAO	-	-	ASH	-

Most patients were asymptomatic (77%, n = 17). Dyspnea and precordial pain were the most frequent symptoms. A familial history of HCM was reported in 64% (n = 17) of subjects. About 59% (n = 13) of the individuals were in use of medications: β-blockers (27%, n = 6); calcium channel blocker (23%, n = 5); and amiodarone (9%, n = 2). Only one patient was in use of more than one medication. The other nine either used no medication or used medications that do not interfere with the presence of late potentials.

**Figure 1:**
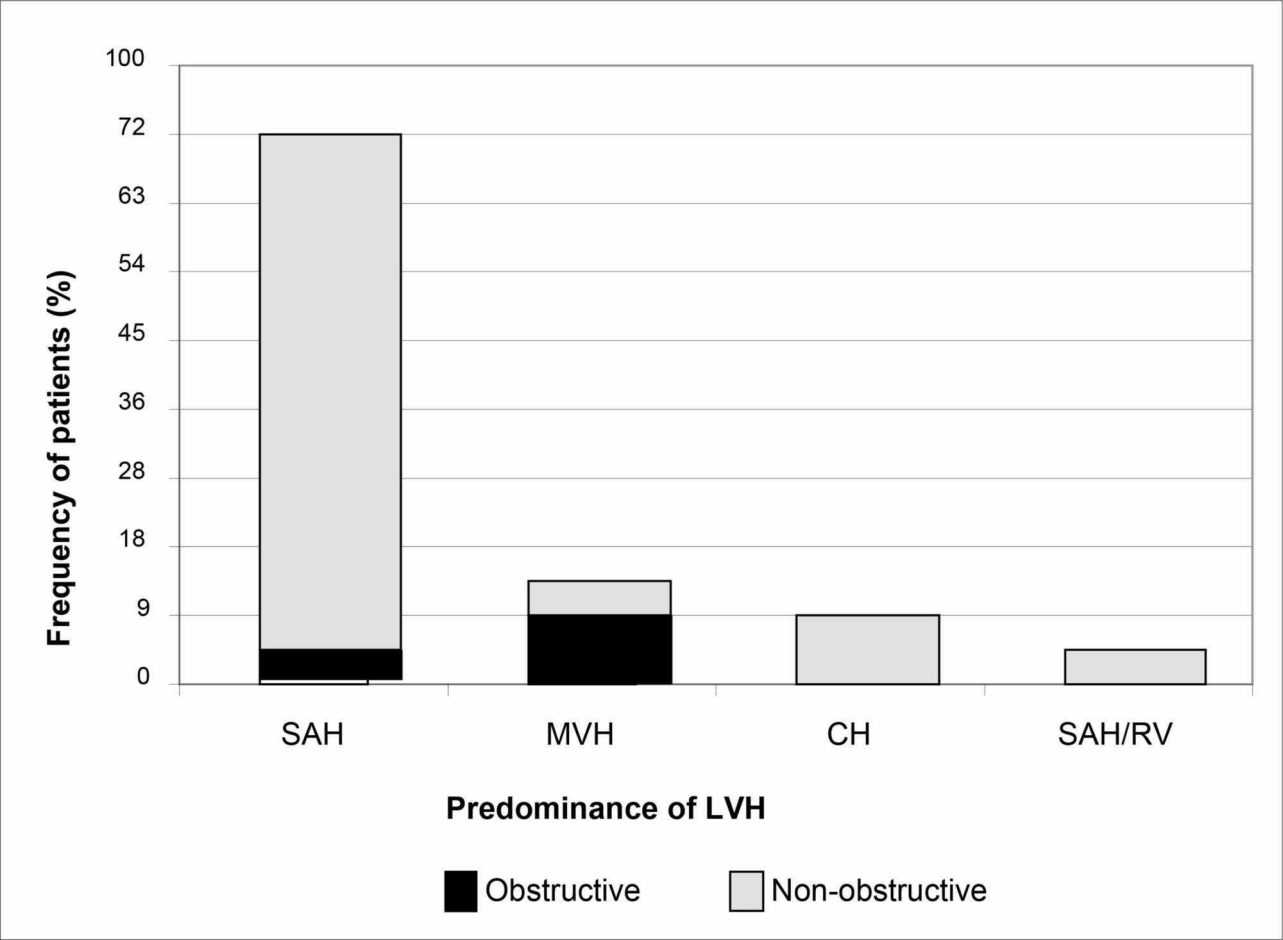
Patients' echocardiographic characteristics SAH: septal asymmetrical hypertrophy; MVH: medium-ventricular hypertrophy; CH: concentric hypertrophy; SAH/RV: septal asymmetrical hypertrophy + right ventricular hypertrophy

Regarding the ventricular hypertrophy predominance on echocardiography, 73% (n = 16) of the individuals presented septal asymmetrical hypertrophy, 9% (n = 2) presented concentric hypertrophy, 14% (n = 3) presented medium-ventricular hypertrophy, and 5% (n = 1) presented septal asymmetrical hypertrophy associated to right ventricular hypertrophy. Regarding the hemodynamic status, the non-obstructive hypertrophy was the most prevalent type (86%, n = 19). Figure 1 summarizes the echocardiographic findings.

Familial sudden death was found in 55% (n = 12) of patients, and 23% (n = 5) of them reported a history of syncope. A total of 23% (n = 5) of the sample had the presence of late potentials in the electrocardiogram. Only in three subjects, both familial sudden death and late potential were present. However, no significant correlation between these variables was found (*p* = 1.00). Syncope and late potentials were associated with two patients, with no significant statistical correlation between them (*p* = 0.54).

Complex ventricular arrhythmias were found in three patients (14%). The concordance between late potentials and complex ventricular arrhythmia was found in only one patient, with no statistical association (*p *= 1.00).

## Discussion

Since Teare et al. reports, HMC has been associated with significant mortality despite major asymptomatic presentation, which justifies the scientific efforts to determine risk factors for sudden death, especially in asymptomatic young subjects [11].

Clinical manifestations are infrequent and might occur at any age [12]. In our sample, the age distribution varied widely from 7 to 54 years. Unlike the reports in the literature, most of our patients were symptomatic (77%), and dyspnea and syncope were the most frequent symptoms. This finding may be explained by the fact that our study was performed in a regional reference center, and symptomatic patients are more likely to be referred for cardiologist evaluation at a tertiary hospital. Nevertheless, only minor changes were described in the physical examination. In HCM patients, the clinical evaluation does not seem to be a reliable tool for diagnosis and risk stratification [12-13]. The electrocardiogram was abnormal in all the patients, of whom 73% presented signs of left ventricular hypertrophy, in accordance with literature descriptions.

We found a prevalence of 64% of a familial history of HCM, and familial sudden death was present in about half of the cases (55%). A recent study conducted by Tobita *et al*. with 52 patients with HCM found that familial history was present in 71.2% and familial history of sudden death in 34.6% of the individuals [3]. History of familial sudden death is more associated with the risk of sudden death than a history of familial HCM only [13-14].

Regarding the pattern of hypertrophy, asymmetric septal hypertrophy was the most common in our sample, corresponding to 73%, which is compatible with previous studies [15]. However, the presence of obstructive hypertrophy was 14%, which is slightly above 25% to 34% described in the literature [12,15]. Obstructive HCM is associated with independent risk for mortality and sudden death and is also correlated with symptomatic manifestations [12-15]. Sudden death might be the initial manifestation in 6% of children and adolescents with HCM, which justify studies to identify risk factors in this population. The presence of familial sudden death, history of syncope, and complex arrhythmias are well-established as significant risk factors. Furthermore, abnormal blood pressure response during upright exercise seems to be a sensitive predictor of risk and is expected mainly in patients with moderate ventricular overflow obstruction [16].

In our sample, late potentials in electrocardiograms were identified in 23% of subjects. Its role in the physiopathology of HCM is still controversial. Nevertheless, some evidence suggests it might be a useful marker for sudden death as it is correlated to an increased risk of fatal arrhythmias [17]. Currently, there are no official recommendations for the use of high-resolution electrocardiography in the risk stratification of HCM patients [18]. Since these shreds of evidence are controversial, we aimed to investigate the association between late potentials and familial sudden death, syncope, and complex ventricular arrhythmias. However, no significant correlation between those variables was demonstrated in our sample.

The implantable cardioverter-defibrillator (ICD) is the most effective therapy in preventing sudden cardiac death. However, in HCM patients, the indications for the implant remain unclear. Primary and secondary sudden death prevention in HCM patients is supported by the European Society of Cardiology Guidelines, especially in patients with a familial history of HCM, unexplained syncope, severe left ventricular hypertrophy, non-sustained tachycardia and history of aborted sudden death [18-19]. Maron et al. demonstrated that the implant was effective in 11% of patients as secondary prevention and 5% as primary prevention in high-risk HCM subjects [12]. In this study, the ICD was implanted in two patients. A significant history of familial sudden death, two episodes of syncope, complex ventricular arrhythmias on Holter and late potentials were present in the patient 6 (Table 1), who was characterized as high risk. The second ICD was indicated in the patient 20 (Table 1), mainly because of his history of familial sudden death in three brothers (at ages one, 16, and 18 years) and report of syncope. Furthermore, this patient also shared similar human leukocyte antigen with one of the dead brothers, which suggests increased genetic risk.

Advances in biomolecular allow the genetic diagnostic of HCM; however, this laboratory approach is not globally available in clinical practice. New studies are needed to determine specific mutations for high-risk individuals. This way, early pharmacological and clinical intervention might reduce the disease progression and the mortality in this population. Nevertheless, in our reality, risk stratification for HCM is expected to be as practical, and low cost as possible, so pharmacological management or ICD implants might be attempted as preventive measures.

The most obvious challenge to conducting rigorous research with rare diseases such as HCM is the small number of eligible participants for a given study, which possibly might be a limitation from our analysis.

## Conclusions

According to this study, the presence of late potentials was not associated with familial sudden death, syncope, and complex ventricular arrhythmias. Familial history of sudden death was present in most of the subjects, which is compatible with the literature description, but complex ventricular arrhythmias and syncope were only observed in a minority of them. On echocardiography, the asymmetric septal hypertrophy was the most common variable, and the non-obstructive form was the hemodynamic pattern most seen.
